# First-In-Human Study on Pharmacokinetics, Safety, and Tolerability of Single and Multiple Escalating Doses of Hepenofovir, a Novel Hepatic Targeting Prodrug of Tenofovir in Healthy Chinese Subjects

**DOI:** 10.3389/fphar.2022.873588

**Published:** 2022-05-19

**Authors:** Hong Zhang, Lei Gao, Jinfeng Lou, Min Wu, Hong Chen, Lizhi Yang, Jingrui Liu, Xiaoxue Zhu, Xiaojiao Li, Cuiyun Li, Meng Wang, Chengjiao Liu, Weibo Guo, Yuan Wang, Zhongqiang Gao, Lei Han, Daidi Wang, Weili Jin, Yanhua Ding

**Affiliations:** ^1^ Phase I Clinical Research Center, The First Hospital of Jilin University, Jilin, China; ^2^ Nanguan District Maternal and Child Health and Family Planning Service Center of Changchun, Jilin, China; ^3^ Xi’an Xintong Pharmaceutical Research Co. Ltd., Xi’an, China

**Keywords:** clinical trial, pharmacokinetics, prodrug, tenofovir, HBV

## Abstract

**Objective:** Hepenofovir, a novel hepatic targeting prodrug of tenofovir, has been developed for the treatment of chronic hepatitis B (CHB). This is a first-in-human study to evaluate the pharmacokinetics (PK) and tolerability of single and multiple escalating doses of hepenofovir in healthy Chinese subjects.

**Methods:** This phase Ia study included two parts: a double-blinded, randomized, placebo-controlled single-ascending-dose (SAD) (25–200 mg) study under fasted conditions comprising a food-effect investigation (200 mg) and a multiple-ascending-dose (MAD) (25 mg) study under fasted conditions.

**Results:** Hepenofovir was well tolerated in healthy Chinese subjects. There was no significant difference in adverse reaction rates between hepenofovir and placebo groups. Hepenofovir was rapidly absorbed and metabolized into tenofovir after dosing. In healthy participants, the median T_max_ of hepenofovir and tenofovir was 0.33–0.50 h and 0.62–0.75 h, respectively, and their mean half-life was 2.5–12.3 h and 49.7–53.8 h, respectively. Systemic exposure to tenofovir increased in proportion to the dose. The mean accumulation indexes of hepenofovir and tenofovir were 1.1 vs. 1.8. Moreover, food could reduce the C_max_ of both hepenofovir and tenofovir, but did not affect their area under the curve (AUC).

**Conclusions:** Hepenofovir has shown a favorable safety and PK profile, which support the further evaluation of its safety and efficacy in CHB patients.

**Clinical trial registration number:** The trial is registered at Chinese Clinical Trial website (http://www.chinadrugtrials.org.cn/index.html # CTR20191953).

## 1 Introduction

Chronic hepatitis B (CHB) virus infection is a global public health issue, which affects more than 400 million people worldwide. The prevalence of CHB is high in Asian and sub-Saharan African in countries (Ott et al., 2012; Spearman et al., 2017). Previous studies have shown that CHB infections can induce cirrhosis and hepatocellular carcinoma (HCC), and ultimately enhance liver-related mortality rates (Fanning et al., 2019). However, HBV treatment has limited success, although nucleoside or nucleotide analogs and peginterferon can effectively inhibit viral replication. It is estimated that there are less than 10% of CHB patients with a loss of hepatitis B surface antigen (HBsAg) and/or seroconversion, which is the ideal endpoint for treating CHB infection (Fanning et al., 2019). Therefore, new potential strategies to treat CHB patients are being explored. Tenofovir disoproxil fumarate (TDF), a nucleotide analogue and potent inhibitor of hepatitis B virus (HBV) polymerase, is one of the promising therapeutic agents for the treatment of CHB infections. The daily TDF dose is 300 mg. However, long-term use of TDF at 300 mg per day might lead to an increased incidence of bone and kidney side effects, such as proximal renal tubulopathy, osteomalacia, or hypophosphatemia (De Clercq, 2016; Viganò et al., 2018; Fong et al., 2019; and; Sutton et al., 2020). It is necessary to develop novel drugs that have higher efficacies with less toxicity to treat CHB infections in a liver-specific manner.

To this end, hepenofovir, a novel hepatic targeting prodrug of tenofovir, has been developed using HepDirect™ patented technology. The chemical formula of hepenofovir is C_18_H_21_ClN_5_O_4_P·C_4_H_4_O_4_ and its formation is described in the formation tablet. Its chemical name is 9-{(2R)-2-[(2R, 4 S)-4-(3- chlorphenyl)-2- oxygen -1,3,2- phosphine dioxane hexane -2- methoxy] propyl} adenine fumaric acid salt. The target active drug is designed to be embedded with an aryl phosphate cyclodiester structure to form the prodrug, which is metabolized into the target active compound by CYP3A4 of cytochrome P450 in the liver, thus achieving its liver targeting effect. Taking advantage of this specific design, the prodrug remains intact in the blood.

Hepenofovir can enhance delivery of tenofovir to the liver, to ensure higher levels of tenofovir in the liver, but lower levels in the plasma, suggesting that hepenofovir would have less renal and bone toxicities compared to TDF. Unlike TDF, the majority of hepenofovir is metabolized to tenofovir primarily by hepatocytes. It maintains its unchanged/inactive form until penetration into the hepatocytes, where hepenofovir is converted to tenofovir by cytochrome P450 isozyme 3A4. Subsequent phosphorylation by cellular enzymes creates tenofovir diphosphate, an active tenofovir metabolite and an obligate chain terminator for HBV. Importantly, this same technology mentioned above has been used in the design of another prodrug, pradefovir, which is currently in phase 3 clinical trials (Lin et al., 2006; Ding et al., 2017; Surial et al., 2020; and; Zhang et al., 2020).

Although there is a certain amount of CY3A4 in intestinal tissue, there is more in the liver. Therefore, the amount of tenofovir in liver tissue is significantly higher compared to intestinal tissue. In a pre-clinical study, the concentration of tenofovir in the liver was six times higher compared to intestinal tissue at 0.25–0.5 h after a single oral administration of hepenofovir (30 mg/kg) in Sprague Dawley rats, which could be maintained only for 24 h, and tenofovir in the intestinal tissue could not be detected 4 h after administration (data not published).

Long-term high concentrations of tenofovir (TDF 300 mg) can cause negative side effects in the kidney and bone, such as proximal renal tubulopathy, osteomalacia, and hypophosphatemia. However, no adverse reactions have been found in other organs and tissues. Still, it is necessary to develop novel liver-targeting drugs (such as hepenofovir in this study) to reduce the levels to tenofovir in the blood, which will in turn reduce the levels of tenofovir in kidney and bone tissue while increasing its concentration in the liver, and improve its efficacy.

The inhibitory effect of hepenofovir on HBV replication was evaluated in a mouse model infected with the adeno-associated virus carrying a replicable hepatitis B virus (AAV/HBV). The results showed that hepenofovir treatment can significantly reduce HBV DNA levels in the serum and liver, with a dose-dependent inhibitory effect that is described in detail as follows. After 3 days of treatment with hepenofovir, the content of HBV DNA in the serum gradually decreased. After 28 days of treatment with hepenofovir in the AAV/HBV mouse model, the contents of HBV DNA in the serum were 1.26, 2.10 and 2.92 log10 copies/ml in the middle or high dose groups (3, 10 and 30 mg/kg, respectively), which were significantly lower than the levels prior to treatment (day 0). After 35 days of treatment, HBV DNA contents were significantly decreased to 0.28, 0.15, 0.38, and 0.51 log10 copies/mg in all treatment groups (1, 3, 10, and 30 mg/kg, respectively) (*p* < 0.05; data not published). The equivalent starting effective dose for humans may be 5–16 mg, according to a conversion using a body surface area method.

Since this study (Phase Ia Clinical Trial of hepenofovir) is the first study to be conducted in China to evaluate its safety profile, tolerability, and pharmacokinetics (PK) in healthy Chinese subjects, the findings of this study will support the Phase Ib Clinical Trial to further evaluate hepenofovir efficacy for treating CHB patients.

## 2 Materials and Methods

### 2.1 Participants

A total of 60 healthy Chinese subjects were enrolled in this study and 59 subjects completed the safety assessment. This is a first-in-human study to evaluate PK, safety, and tolerability of single and multiple escalating doses of hepenofovir, in the presence or absence of food. The main inclusion criteria included: age, 18–55 years; body mass index (BMI), 18–28 kg/m^2^; without clinically relevant condition, according to physical examination results; and with eligible laboratory test results, electrocardiography, and medical history. The main exclusion criteria included: use of alcohol or a drug that is known to influence cytochrome P450 or affect gastric pH within 2 weeks before dosing; and pregnant or breastfeeding women.

### 2.2 Study Design

**FIGURE 1:**
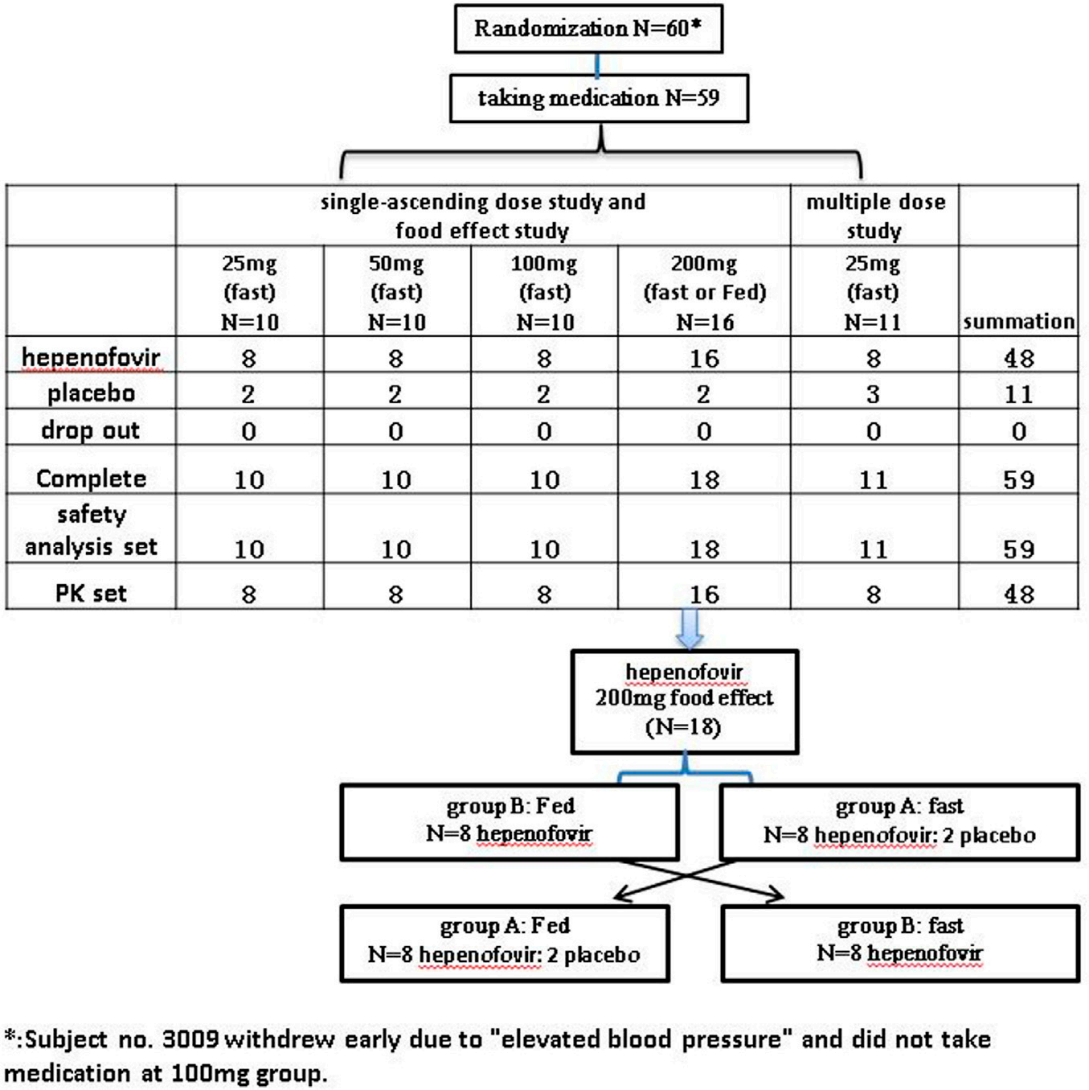
Flow-chart depicting the variables examined in the study.

This was a randomized, double-blind, placebo-controlled, and ascending-dose study (Chinese Drug Trial Identifier: CTR20191953). The protocol of this clinical trial was approved by the Ethics Committee of The First Hospital of Jilin University (Changchun, Jilin, China). Our study was conducted in agreement with the guidelines of the Declaration of Helsinki and the International Conference on Harmonization Good Clinical Practice Guidelines and fulfilled local regulatory requirements. All subjects recruited for this study provided written informed consent prior to their enrollment. The flow-chart of the present study is shown in Figure 1.

#### Single Ascending Dose Cohort and Food Effect

Forty-eight healthy subjects were randomly assigned to five single dose groups (0, 25, 50, 100 or 200 mg), as shown in the Supplementary Materials. Each group contained 10 subjects (hepenofovir:placebo = 8:2) who received the drug under fasted conditions, except for the 200 mg group. Based on the adverse event reports, vital sign evaluation, as well as the results of clinical laboratory tests, 12-lead electrocardiogram, and physical examination, the tolerability of hepenofovir was assessed on day three and day six. The hepenofovir treatment was initiated in the group receiving a single dose of 25 mg. If safety and tolerability were confirmed in the 25 mg group, the subsequent increased dose was investigated, and so on.

The effect of food on the PK of hepenofovir was observed in the 200 mg group (18 subjects; 16:2 for hepenofovir:placebo), in a single-dose, randomized, two-period, two-sequence crossover study under fasted or fed conditions (Figure 1). The metabolism of hepenofovir was examined in the fasted condition using fecal and urine samples. In the food effect study, subjects consumed a high fat and high calorie food (high-fat: approximately 50% of total caloric content of the meal; high calorie: approximately 800–1000 kcal) approximately 30 min before drug administration. The washout time was 14 days. Hepenofovir tolerability was evaluated after dosing on days 3, 6, 17, and 20.

#### Multiple-Dose Cohort

Initially, 11 healthy subjects (8:3 for hepenofovir:placebo) were randomly assigned to receive hepenofovir (25 mg) once daily for 7 days, to evaluate the safety and tolerability at 25 mg. In this multiple-dose study, tolerability was assessed after dosing on days 3, 6, and 12, under fasted conditions.

Next, sentinel dosing was adopted for the evaluation of the ascending dose. Two subjects were enrolled in one of the SAD groups (including 25, 50, 100 and 200 mg) as well as a multiple-dose group (all received hepenofovir, one male and one female subject). After observation for 48 h in the SAD study or 72 h in the multiple-dose study, the remaining eight or nine subjects were enrolled if the first two subjects revealed that the drug was safe and well tolerated.

All treatment groups were balanced regarding their demographic and baseline characteristics (Table 1). It was required for subjects to fast longer than 10 h prior to the collection of their blood samples for clinical laboratory tests. The subjects were required to comply with the guidelines of the Phase I Clinical Research Center at the First Hospital of Jilin University, which included regulation of diet and activity. The investigational drug was orally administered while subjects drank 240 ml of water. Both hepenofovir and placebo were synthesized and provided by Xi’an Xintong Research Co. Ltd. The formulation of both hepenofovir and its placebo was a tablet, and the size of the drug was 12.5 and 50 mg (lot #190602/190601 and #190604/190603), respectively.

**TABLE 1:** Demographic characteristics of participants.

Baseline parameter	Placebo (n = 11)	Single-ascending dose study and food effect study					Multiple-dose study
		25 mg (n = 8)	50 mg (n = 8)	100 mg (n = 9^a^)	200 mg (group A) (n = 8)	200 mg (group B) (n = 8)	25 mg (n = 8)
Age in years, mean (SD)	36.9 (7.4)	33.9 (7.9)	36.8 (9.5)	37.2 (9.1)	41.3 (7.5)	32.6 (7.7)	38.1 (6.7)
Sex (male/female)	5/6	4/4	4/4	5/4	4/4	4/4	4/4
Ethnic (Han/Man)	11/0	8/0	6/2	9/0	8/0	8/0	8/0
BMI (kg/m2), mean (SD)	22.9 (2.3)	21.9 (2.5)	23.0 (2.7)	22.8 (2.7)	25.0 (2.7)	23.8 (2.7)	23.6 (2.4)

a Subject no. 3009 withdrew early due to “elevated blood pressure” and did not take medication at 100 mg group. Abbreviations: BMI, body mass index; SD, standard deviation.

### 2.3 Pharmacokinetics Analysis

Venous blood (4 ml) was collected and then added to a tube containing K2EDTA as an anticoagulant. After centrifugation (1500 x *g* at 2–8°C for 10 min), plasma was collected and stored at −80°C until used for PK analysis. The detailed time points of blood collection are provided with the supplementary material.

Several PK parameters were analyzed in this study, including the maximum observed plasma concentration (C_max_), terminal elimination half-life of the drug in plasma (t_½_), time to maximum observed plasma concentration (T_max_), area under the concentration-time curve (AUC) from time of dosing (0 h) to 120 h (AUC_0–120h_), and the AUC from time of dosing extrapolated to infinity (AUC_0-∞_), which were assessed at first dose. Plasma samples were also collected to measure the following PK parameters at steady state (ss), such as C_ss max_, t_ss__½_, T_ss max_, AUC_ss 0–120 h_, accumulation index, and degree of fluctuation at last dose. The plasma concentration versus time curve of hepenofovir was analyzed using non-compartmental methods with WinNonlin Professional software (version 6.4, Pharsight Corporation, NC, United State), of which the linear trapezoidal method was used for calculating the AUC.

#### For Single Ascending Dose Study With Ascending Doses: 25, 50, 100, or 200 mg Hepenofovir

Collection of 4 ml venous blood was performed from 30 min prior to dosing up to 120 h post-dosing. In the food effect group (with 200 mg hepenofovir), blood was collected on day 1 and day 15, which was the same procedure as the SAD group. Urine samples were also collected at 0 h pre-dosing as well as 0–6, 6–12, 12–24, 24–48, 48–72, 72–96 and 96–120 h post-dosing on day 15, from subjects in the 200 mg hepenofovir group B (under fasting conditions). Fecal samples were also collected from these same subjects within 120 h after dosing on day 15.

#### Multiple-Dose Study With 25 mg

Eleven healthy subjects (8:3 for hepenofovir:placebo) were randomly assigned to receive hepenofovir (25 mg) once daily for 7 days. Their blood samples were collected from 30 min prior to dosing to 24 h after dosing on day 1, and from 30 min prior to dosing up to 120 h after dosing on day 7, and 0–30 min before dosing on days 4 through 6.

### 2.4 The Concentration of Hepenofovir and Tenofovir in Biological Samples

The plasma, urine, and fecal concentrations of hepenofovir and tenofovir were measured by Frontage Laboratories Inc. (Shanghai, China) using high-pressure liquid chromatography (HPLC) and tandem mass spectrometry (MS/MS). All collected plasma samples were within the known period of stability, and the quality met pre-established acceptance criteria. The determination of the plasma drug concentration was conducted in compliance with applicable standard operating procedures. The standard curve ranges for both hepenofovir and tenofovir were 0.3–500 ng/ml in the plasma; 25–25,000 ng/ml in the urine; and 2.5–2500 ng/g in the fecal samples. The measurement accuracies for the plasma, urine, and fecal samples ranged from −2 to 0.3%, -0.8–4.2%, and −3 to 1.3%, respectively. The intra- and inter-day precision for the measurement of the plasma, urine, and fecal samples ranged from 0 to 5.2%, 1.3–5.4%, and 1.4–14.1%, respectively, as measured by the coefficient of variation.

### 2.5 Statistical Analysis

Several descriptive statistical methods were used for the comparisons of PK parameters of hepenofovir and tenofovir. The analysis of variance (ANOVA) was used with factors fitted for the effect of subject and the period as fixed effects to compare C_max_ and AUC in the food effect study. The comparison results were shown as geometric least-square means with the 90% confidence interval (CI). SAS 9.4 software was used to determine the dose proportionality relationship (25–200 mg) and the Cmax and AUC of hepenofovir and tenofovir were determined using the Linear Mixed Effect Model. The dose and exposure levels were fitted after taking natural logarithms. The fitting formula is as follows: Ln (PK) = β0+β1*Ln (dose). The statistical analysis results are presented as the mean (standard deviation).

## 3 Results

### 3.1 Demographics

The age range of the participants in each group was 32.6–41.3 years. The body mass index (BMI) range of subjects in each group was 21.9–25.0 kg/m^2^, with similar sex ratios (1:1 male/female ratio). Almost all participants were Han Chinese (96.7%). The demographic data were balanced among the different groups. All participants completed this study, as shown in Table 1.

### 3.2 Pharmacokinetics Profiles of Hepenofovir and Tenofovir

#### 3.2.1 Single Ascending Dose Group

This study found that hepenofovir could be rapidly absorbed and metabolized into tenofovir after a single oral administration. In healthy subjects, the median T_max_ of either hepenofovir or tenofovir was 0.33–0.50 h and 0.62–0.75 h, respectively, with a declining trend resembling a two-compartment model that shows a rapid first distribution, followed by a slow elimination when subjects were in a fasted state.

**FIGURE 2:**
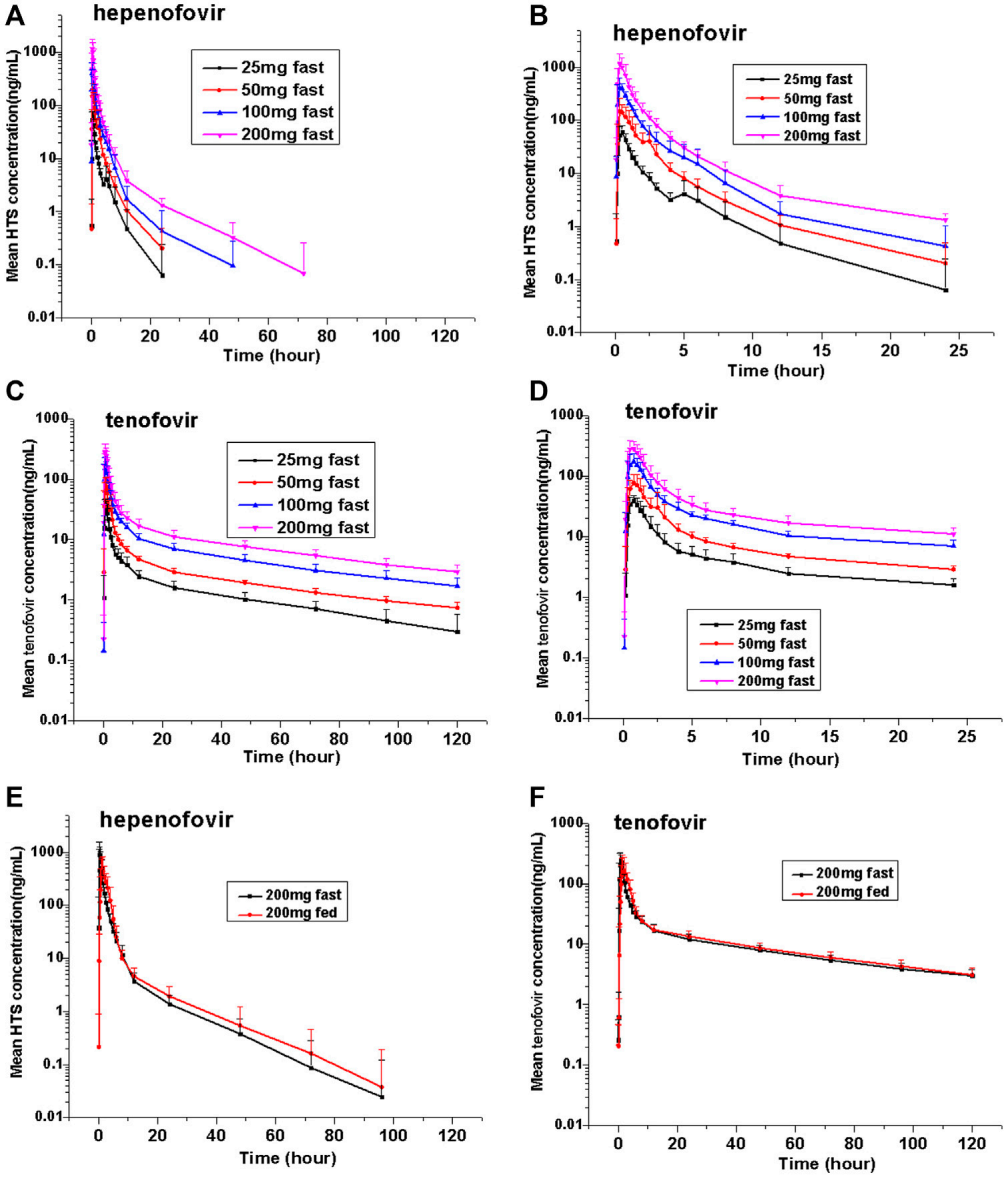
Mean values of plasma hepenofovir and tenofovir concentration-time profiles in each treatment group for the single-ascending dose study and food effect study. Data illustrations included: mean (± standard deviation [SD]) hepenofovir plasma concentration-time profiles **(A)** and mean (±SD) hepenofovir plasma concentration-time of 0–24 h profiles **(B)**; mean (±SD) tenofovir plasma concentration-time profiles **(C)** and mean (±S **(D)** tenofovir plasma concentration-time of 0–24 h profiles **(D)**; mean (±SD) hepenofovir plasma concentration-time profiles of the food effect study **(E)**; mean (±SD) tenofovir plasma concentration-time profiles of the food effect study **(F)**.

The exposure (C_max_ and AUC) of both hepenofovir and tenofovir increased with ascending dosages. The clearance of hepenofovir decreased with its increased dosage. The volume of hepenofovir distribution also increased with its increased dosage, and the mean t_1/2_ of hepenofovir (range: 2.5–12.3 h) increased with its increased dosage. However, compared to that of hepenofovir, the mean t_1/2_ of tenofovir was relatively long, and its clearance and volume of distribution were similar between the different dosage groups of tenofovir, resulting in a similar t_1/2_, ranging from 49.7 to 53.8 h (Table 2 Figure 2, Supplementary Table 1).

**TABLE 2:** Pharmacokinetic parameters of tenofovir of the single-dose in each treatment group in the fasted state (mean ± SD).

PK parameters	25 mg (n = 8)	50 mg (n = 8)	100 mg (n = 8)	200 mg (n = 8)
^a^T_max_ (h)	0.62 (0.50, 1.00)	0.75 (0.50, 2.50)	0.75 (0.33, 1.00)	0.62 (0.50, 1.00)
C_max_ (ng/ml)	41 (8)	92 (22)	186 (60)	320 (69)
AUC_0–24h_ (h*ng/mL)	121 (34)	245 (37)	540 (116)	854 (261)
AUC_0–120h_ (h*ng/mL)	194 (59)	390 (51)	882 (189)	1429 (394)
AUC_0-∞_ (h*ng/mL)	230 (82)	450 (66)	1017 (232)	1662 (481)
t_1/2_ (h)	49.7 (16.4)	53.8 (11.7)	53.6 (11.8)	53.8 (6.4)
AUC__%Extrap_ (%)	14.56 (5.30)	13.04 (4.52)	12.98 (4.64)	13.78 (2.54)

a Median (min-max). PK, pharmacokinetic; SD, standard deviation.

The values of the geometric means of the AUC and C_max_ of hepenofovir ranging from 25 to 200 mg showed a slight non-linear increase (an increase with more than proportion to dose) (Smith et al., 2000). The regression coefficients of the power model for either C_max_ or AUC were 1.30 or 1.47, respectively. However, the values of the geometric means of the AUC and C_max_ of tenofovir in different dosage groups, ranging from 25 to 200 mg, increased linearly (increase in nearly direct proportion to dose), and its regression coefficients of the power model for C_max_ and AUC were 0.95 or 0.98, respectively (Supplementary Table 2).

#### 3.2.2 Food Effect Study

In the food effect study, the administration of 200 mg hepenofovir with food resulted in lower plasma C_max_ levels (53.6% for hepenofovir, and 95.0% for tenofovir) while there were similar plasma AUC values between the fasted and fed groups. Moreover, compared to the fasting condition, drug absorption was delayed when food was consumed prior to drug administration, and the median T_max_ was delayed for 1.0 h. The PK profile in the elimination phase was similar between the fasted and fed groups (Figure 2, Supplementary Table 3).

After a single oral administration of 200 mg hepenofovir in eight healthy subjects in a fasted state, the mean cumulative excretion of hepenofovir in the urine or feces within 120 h (sample collection up to 120 h) was 17092480.4 ng (approximately 17 mg) and 6987638.3 ng (approximately 7 mg), respectively. The mean cumulative excretion of hepenofovir was 8.54% (urine) and 3.49% (feces). On the other hand, the average cumulative excretion of tenofovir in the urine or feces within 120 h (sample collection up to 120 h) was 13660381.5 ng (approximately 14 mg) and 12436749.1 ng (approximately 12 mg), respectively. The average cumulative excretion of tenofovir was 13.17% (urine) and 11.99% (feces).

#### 3.2.3 Multiple-Dose Group

Similar to the results of the SAD group, hepenofovir was also rapidly absorbed and metabolized into tenofovir in the multiple-dose group. The median T_max_ of hepenofovir or tenofovir in the multiple-dose group was 0.41–0.50 h and 0.62–0.87 h, respectively. The mean t_1/2_ of both drugs was 2.6–3.7 h and 17.6–57.4 h, respectively.

**FIGURE 3:**
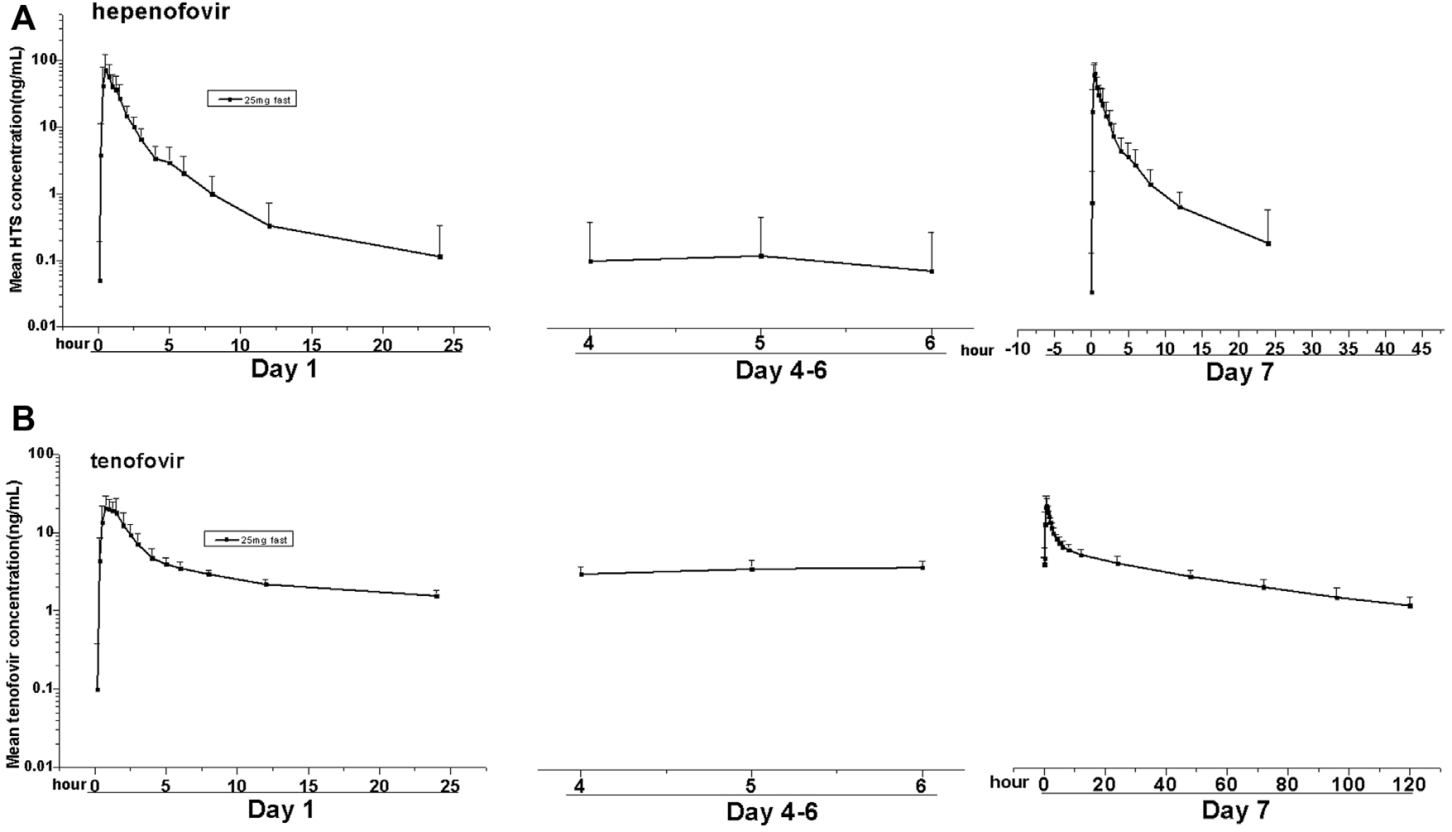
Mean values of plasma hepenofovir and tenofovir concentration-time profiles in each treatment group for the multiple-dose study. Data illustrations included: mean (± standard deviation [SD]) hepenofovir plasma concentration-time profiles **(A)** and tenofovir plasma concentration-time profiles **(B)**.

After multiple administrations, the exposure amount of hepenofovir with a short half-life was similar to that after a single administration, resulting in very little accumulation. Results showed an hepenofovir accumulation index of 1.1, based on the ratio of AUC day 7 versus AUC day 1 with a large fluctuation (1970%). In comparison, tenofovir had a longer half-life, thus it also had a longer exposure and a cumulative index of 1.8 (day 7 AUC versus day 1 AUC) with a relatively small fluctuation (305%). Taken together, the accumulation index was small for both hepenofovir and its metabolite tenofovir, indicating negligible accumulation after steady state, since hepenofovir and its metabolite tenofovir reached a steady state after 4 days of administration (Figure 3, Table 3, Supplementary Table 4).

**TABLE 3:** Pharmacokinetic parameters of tenofovir of the multiple-dose 25 mg group (mean ± SD; n = 8).

PK parameters	Day 1	Day 7
^a^T_max_ (h)	0.87 (0.75, 1.50)	0.62 (0.50, 1.50)
C_max_ (ng/ml)	25 (9)	25 (6)
AUC_0–24h_ (h*ng/mL)	91 (20)	164 (28)
AUC_0–120h_ (h*ng/mL)	90 (19)	384 (78)
AUC_0-∞_ (h*ng/mL)	131 (34)	487 (116)
t_1/2_ (h)	17.6 (5.1)	57.4 (8.4)
RAC (AUC)	--	1.8 (0.3)
Fluctuation (%)	--	305 (64)
AUC__%Extrap_ (%)	30.24 (7.30)	20.54 (3.85)

a Median (min-max).

### 3.3 Safety and Tolerability

All 59 subjects who completed this study were included in the safety analysis. Results showed that the drug was well tolerated after administration, without serious adverse reactions or adverse reactions that caused withdrawal, and without severe adverse reactions of grade III or above (CTC-AE version 5.0). Fourteen events of adverse reactions were “grade I” and three events of adverse reactions (hypertriglyceridemia) were “grade II”.

In this study, the incidence of adverse reactions was 18.2% (two cases/four times) in the placebo group; 25.0% (two cases/two times) in the 25 mg single dose group, 37.5% (three cases/three times) in the 50 mg single dose group; 12.5% (one case/one time) in the 100 mg single dose group; 25.0% (two cases/three times) in the 200 mg Group A; 37.5% (three cases/three times) in the 200 mg Group B; and 12.5% (one case/one time) in the 25 mg multiple-dose group. From the above information, the incidence of adverse reactions did not increase significantly as the dose was increased, and there was no statistical difference in the incidence of adverse reactions among the different groups (*p* = 0.6937).

The most common adverse reactions of hepenofovir were hypokalemia, hypophosphatemia, hematuria, hyperglycemia, increased creatinine, hypertriglyceridemia, increased urinary white blood cell count, and hyperuricemia, with a low incidence of 12.5% within each group. Oral administration of hepenofovir at doses ranging from 25 to 200 mg was safe and tolerated in healthy Chinese subjects (Table 4).

**TABLE 4:** Treatment-emergent adverse reactions.

	Placebo (n = 11)	Single-ascending dose study and food effect study					Multiple-dose study
		25 mg (n = 8)	50 mg (n = 8)	100 mg (n = 8)	200 mg (group A) (n = 8)	200 mg (group B) (n = 8)	25 mg (n = 8)
Total	2 (18.2)	2 (25)	3 (37.5)	1 (12.5)	2 (25)	3 (37.5)	1 (12.5)
Hypokalemia	0 (0)	1 (12.5)	0 (0)	0 (0)	0 (0)	0 (0)	0 (0)
Hypophosphatemia	0 (0)	1 (12.5)	1 (12.5)	0 (0)	0 (0)	1 (12.5)	0 (0)
Hypertriglyceridemia	0 (0)	0 (0)	0 (0)	0 (0)	1 (12.5)	0 (0)	1 (12.5)
Hyperuricemia	0 (0)	0 (0)	0 (0)	0 (0)	0 (0)	1 (12.5)	0 (0)
Hyperglycemia	0 (0)	0 (0)	1 (12.5)	0 (0)	1 (12.5)	0 (0)	0 (0)
Elevated alanine aminotransferase	1 (9.1)	0 (0)	0 (0)	0 (0)	0 (0)	0 (0)	0 (0)
Urine leucocyte positive	1 (9.1)	0 (0)	0 (0)	0 (0)	0 (0)	1 (12.5)	0 (0)
Uroerythropoiesis	1 (9.1)	0 (0)	0 (0)	0 (0)	0 (0)	0 (0)	0 (0)
Elevated serum creatinine	1 (9.1)	0 (0)	0 (0)	1 (12.5)	0 (0)	0 (0)	0 (0)
Hematuria	0 (0)	0 (0)	1 (12.5)	0 (0)	0 (0)	0 (0)	0 (0)

The data are presented as n (%).

## 4 Discussion

Our study showed that hepenofovir is tolerated in healthy Chinese subjects, and no serious adverse reactions occurred during the period of the clinical trial that would have led to its discontinuation. As we know, tenofovir is primarily eliminated by the kidneys. It has been reported that Tenofovir can cause proximal renal tubulopathy in some patients who receive tenofovir dipyoproxil fumarate tablets (300 mg), due to the higher exposure to tenofovir in the kidneys, leading to an increase in creatinine levels and a decrease in glomerular filtration rate, eventually causing hypophosphatemia and osteoporosis (Lampertico et al., 2020; Lee et al., 2021; Nguyen et al., 2021). Liver-targeting prodrugs have been developed since they can increase the concentration of active molecular components of a specific drug in the liver, while significantly reducing the plasma concentration (and therefore kidney exposure), resulting in improved efficacy of the drug in the target tissue, with less toxicity in non-target tissues (Chan et al., 2016; Lampertico et al., 2020).

In this study, there were two subjects (one in the placebo group and one in the hepenofovir 100 mg group) who had increased serum creatinine levels (severity: grade I). The blood phosphorus levels of three subjects given different doses of hepenofovir (25, 50 and 200 mg) were found to be decreased (severity: grade I). These changes recovered without intervention. However, the exposure to hepenofovir in thoee subjects was even lower compared to the other subjects in each group. Therefore, the incidence of adverse reactions might not be correlated with exposure (Table 2). All the above-mentioned adverse reactions occurred in different subjects, which is not consistent with the chronological sequence of kidney injury caused by tenofovir. Meanwhile, these possible drug-related adverse reactions are characterized with low incidence, without a dose-dependent relationship, and recovering without intervention by the end of the trial. Whether hepenofovir has adverse reactions related to long-term renal injury and decreased phosphorus remains unclear. Further investigation is needed after long-term dosing. The safety and tolerability of hepenofovir are consistent with those of tenofovir alafenamide (TAF, a new phosphonate prodrug of tenofovir) in clinical studies (Agarwal et al., 2015; Yamada et al., 2019; and; Li et al., 2021).

In this study, the PK profiles of hepenofovir and its metabolite tenofovir were investigated in healthy subjects, which resembled a two-compartment model with first-order (two-stage) absorption (Zhang et al., 2021a). The study showed rapid oral absorption and metabolism of hepenofovir. In the hepenofovir group, the C_max_ and AUC values increased, with a proportion slightly greater than the dose. However, the values of C_max_ and AUC for tenofovir increased linearly in proportion to the dose. The differences in C_max_ and AUC between hepenofovir and tenofovir might be explained by the following. Hepenofovir is a liver-targeting prodrug of tenofovir, where the exposure to hepenofovir may increase to reach the maximum capacity of liver cells to metabolize hepenofovir. As a result, the exposure to hepenofovir in the plasma also increased, which was more than the dosage proportion (de Campos et al., 2014; and; Zhang et al., 2021b).

On the other hand, tenofovir is characterized by a quick elimination in the initial stage and a slow elimination in the low concentration stage. When calculating PK parameters over a longer period, such as increasing 0–24 h to 0–120 h, the calculated t_1/2_ value will be longer (Zhang et al., 2021a). Therefore, after multiple-doses of hepenofovir, a longer t_1/2_ and a large difference in t_1/2_ between hepenofovir and tenofovir were found. This study also revealed that the administration of hepenofovir in the presence of food did not influence the AUC, but did decrease C_max_, which suggests no requirement to consider the effects of food on the efficacy of hepenofovir therapy (Karalis et al., 2008; and; Zhang et al., 2018). In greater detail, the AUC% extrapolation was less than 20%, indicating that the collection of time points for the analysis of PK parameters was sufficient. Since the multiple-dose cohort required continuous dosing, the collection time could only be 30 min prior to dosing to 24 h after dosing on day 1, resulting in a relatively large AUC% extrapolation (30.24%) at day 1 for tenofovir.

The plasma exposure level of tenofovir in the 25–200 mg hepenofovir groups was lower than that in the 300 mg TDF group. Even at the same dose, for example, the AUC_0–48 h_ of tenofovir in the TDF 200 mg group (1312 h*ng/mL) was higher compared to the hepenofovir 200 mg group (1047 h*ng/mL) (Yerino et al., 2011), demonstrating the possible liver-targeting feature of hepenofovir. Moreover, the equivalent starting effective dose for humans may be 5–16 mg, based on the body surface area method for conversion and calculation using data from the pre-clinical study. Thereby, it is recommended to use approximately 25–200 mg of hepenofovir to treat CHB patients in the Phase Ib Clinical Study.

## 5 Conclusion

This study demonstrated a safe and tolerable profile of hepenofovir with a potent hepatic targeting feature to provide useful information in support of future clinical trials to further evaluate its safety and efficacy for the treatment of CHB patients.

## Data Availability

The original contributions presented in the study are included in the article/Supplementary Material, further inquiries can be directed to the corresponding author.
