# Supplementary material for: First-In-Human Study on Pharmacokinetics, Safety, and Tolerability of Single and Multiple Escalating Doses of Hepenofovir, a Novel Hepatic Targeting Prodrug of Tenofovir in Healthy Chinese Subjects
Source: Front Pharmacol. 2022 May 19;13:873588. doi: 10.3389/fphar.2022.873588 (PMC9161552; doi:10.3389/fphar.2022.873588)
Supplement: Supplementary file 1 [file Table1.docx]

**Supplementary Methods**

According to the food effect study guidelines, when a drug will be marketed and several doses exhibit a linear PK, the sponsor should use the highest clinically recommended dose unless safety concerns necessitate a lower dose.

In preclinical studies, the No Observed Adverse Effect Level (NOAEL) of hepenofovir was 100 mg/kg for Sprague Dawley (SD) rats, and the NOAEL of [machin](javascript:;) was 15 mg/kg. The recommended maximum starting dose for humans may be 36-121 mg, according to a conversion calculation using a method involving the body surface area with a scale factor of 10. Therefore, the single ascending dose groups were decided to be 0, 25, 50, 100, 200, 300 mg, and the effect of food on the PK of hepenofovir was observed in the 200 mg group.

Following observation of the 25-200 mg groups (including the food effect study), an overall analysis of tolerability and PK was performed using the obtained data. We found that the 25-200 mg groups covered the dose range for future phase II-III clinical studies, and the 300 mg group was terminated.

Finally, the food effect study was completed at the highest clinical dose in this study.

***Collection time points for analysis of PK parameters***

Collection of blood samples for analysis of PK profiles for both hepenofovir and tenofovir was performed at the following time points. Once the concentration of the previous group was measured, the sampling points of the latter group could be adjusted, according to the concentration of the previous group.

In the SAD groups with 25, 50, or 100 mg of hepenofovir, collection time points included 30 min before dosing and 5 min, 10 min, 20 min, 30 min, 45 min, 1 h, 1.25 h, 1.5 h, 2 h, 2.5 h, 3 h, 4 h, 5 h, 6 h, 8 h, 12 h, 24 h, 48 h, 72 h, 96 h, and 120 h after dosing on day 1.

In the SAD group with 200 mg hepenofovir, collections were performed at 30 min before dosing, and at 5 min, 10 min, 20 min, 30 min, 45 min, 1 h, 1.25 h, 1.5 h, 2 h, 2.5 h, 3 h, 4 h, 5 h, 6 h, 8 h, 12 h, 24 h, 48 h, 72 h, 96 h, and 120 h after dosing on day 1 and day 15.

In the multiple-dose group with 25 mg hepenofovir, collections were performed 30 min before dosing, and 5 min, 10 min, 20 min, 30 min, 45 min, 1 h, 1.25 h, 1.5 h, 2 h, 2.5 h, 3 h, 4 h, 5 h, 6 h, 8 h, 12 h, and 24h on day 1, and ≤ 30 min before dosing on days 4 through 6, as well as 30 min before dosing, and 5 min, 10 min, 20 min, 30 min, 45 min, 1 h, 1.25 h, 1.5 h, 2 h, 2.5 h, 3 h, 4 h, 5 h, 6 h, 8 h, 12 h, 24 h, 48 h, 72 h, 96 h, and 120 h on day 7.

The parameters for hepenofovir analysis: The [charge-to-mass](javascript:;) [ratio](javascript:;) (m/z) is 438.1/151.1, and mobile phase include Mobile phase A: aqueous solution containing 0.1% formic acid and 10mM ammonium acetate; Mobile phase B: a 50/50 mixture of acetonitrile and methanol; Flow rate: 0.4mL/min. The column is ACE Excel 3 C18 50*2.1mm, 3 micron.

The parameters for tenofovir analysis: The m/z is 288.2/176.2, and mobile phase include Mobile phase A: aqueous solution containing 0.6% formic acid and 10mM ammonium formate, mixed in a ratio of 5/95 by volume; Mobile phase B: methanol; Flow rate: 1.0 mL/min. The column is Waters Xbridge® C18 50×4.6 mm, 3.5 micron.

**Supplementary** **Table 1. Pharmacokinetic parameters of hepenofovir in each single-dose treatment group (mean ± SD).**

| PK parameters | 25 mg (n = 8) | 50 mg (n = 8) | 100 mg (n = 8) | 200 mg (n = 8) |
| --- | --- | --- | --- | --- |
| ^#^T_max_ (h) | 0.50 (0.33, 0.75) | 0.41 (0.33, 2.50) | 0.41 (0.33, 0.50) | 0.33 (0.17, 0.75) |
| C_max_ (ng/mL) | 64 (17) | 193 (84) | 478 (169) | 1406 (458) |
| AUC_0-24h_ (h*ng/mL) | 85 (22) | 250 (74) | 587 (147) | 1288 (388) |
| AUC_0-120h_ (h*ng/mL) | 83 (22) | 248 (75) | 590 (148) | 1303 (385) |
| AUC_0-∞_ (h*ng/mL) | 86 (23) | 252 (74) | 594 (148) | 1314 (383) |
| t_1/2_ (h) | 2.5 (1.4) | 3.2 (1.8) | 5.0 (5.1) | 12.3 (9.4) |
| AUC__%Extrap_ (%) | 2.69 (1.14) | 1.72 (1.31) | 0.64 (0.41) | 0.92 (0.58) |

^#^: median (min-max).

**Supplementary Table 2. Analysis of the** **dose proportionality of hepenofovir and tenofovir with power model**

| PK parameter | [Regression](javascript:;) [coefficient](javascript:;) | 90% CI |
| --- | --- | --- |
| **Hepenofovir** | | |
| C_max_ (ng/mL) | 1.47 | 1.35 - 1.59 |
| AUC_0-24h_ (h*ng/mL) | 1.30 | 1.19 - 1.40 |
| AUC_0-120h_ (h*ng/mL) | 1.31 | 1.21 - 1.42 |
| AUC_0-∞_ (h*ng/mL) | 1.30 | 1.20 - 1.41 |
| **Tenofovir** | | |
| C_max_ (ng/mL) | 0.98 | 0.88 - 1.08 |
| AUC_0-24h_ (h*ng/mL) | 0.95 | 0.86 - 1.05 |
| AUC_0-120h_ (h*ng/mL) | 0.98 | 0.88 - 1.08 |
| AUC_0-∞_ (h*ng/mL) | 0.97 | 0.87 - 1.08 |

**Supplementary Table 3. Analysis of pharmacokinetic effects of high-fat food.**

|  | **Hepenofovir** | | | **Tenofovir** | | |
| --- | --- | --- | --- | --- | --- | --- |
| **PK parameter** | **Fed** | **Fasted** | **Ratio (fed versus fast, %)** | **Fed** | **Fasted** | **Ratio (fed versus fast, %)** |
| C_max_(ng/mL) | 678 (300) | 1251 (539) | 53.6 (43.1 - 66.7) | 235 (106) | 277 (71) | 95.0 (85.4 - 105.8) |
| AUC_0-24h_(h*ng/mL) | 1203 (365) | 1270 (320) | 93.5 (84.4 - 103.7) | 852 (225) | 824 (195) | 95.0 (85.4 - 105.8) |
| AUC_0-120h_ (h*ng/mL) | 1231 (378) | 1290 (317) | 94.0 (84.7 - 104.4) | 1506 (351) | 1419 (294) | 95.0 (85.4 - 105.8) |
| AUC_0-∞_(h*ng/mL) | 1253 (377) | 1300 (316) | 95.0 (85.4 - 105.8) | 1737 (422) | 1649 (362) | 95.0 (85.4 - 105.8) |
| ^#^T_max_(h) | 1.50 (0.50, 3.00) | 0.50 (0.17, 1.00) |  | 1.75 (1.00, 4.00) | 0.87 (0.50, 1.25) |  |
| t_1/2_(h) | 15.9 (15.0) | 12.6 (8.7) |  | 49.8 (10.0) | 51.6 (8.1) |  |

^#^: median (min-max).

**Supplementary** **Table 4. Pharmacokinetic parameters of hepenofovir in the 25 mg multiple-dose group (mean ± SD, n = 8).**

| PK Parameters | Day 1 | Day 7 |
| --- | --- | --- |
| ^#^T_max_ (h) | 0.50 (0.33, 1.25) | 0.41 (0.33, 1.50) |
| C_max_ (ng/mL) | 93 (44) | 85 (21) |
| AUC_0-24h_ (h*ng/mL) | 99 (33) | 110 (45) |
| AUC_0-120h_ (h*ng/mL) | 98 (33) | 108 (45) |
| AUC_0-∞_ (h*ng/mL) | 100 (34) | 112 (48) |
| t_1/2_ (h) | 2.6 (1.4) | 3.7 (1.9) |
| RAC (AUC) | -- | 1.1 (0.2) |
| Fluctuation (%) | -- | 1970 (454) |
| AUC__%Extrap_ (%) | 1.74 (0.53) | 2.87 (1.51) |

^#^: median (min-max).
